# Protein X-ray crystallography of the 14-3-3ζ/SOS1 complex

**DOI:** 10.1016/j.dib.2018.06.060

**Published:** 2018-06-28

**Authors:** Alice Ballone, Federica Centorrino, Madita Wolter, Christian Ottmann

**Affiliations:** a Department of Biomedical Engineering, Eindhoven University of Technology, Laboratory of Chemical Biology, Eindhoven, 5600 MB, The Netherlands; b Department of Chemistry, University of Duisburg-Essen, Universitätsstr. 7, 45117 Essen, Germany

## Abstract

Activation of Ras-MAPK signaling regulates essential cellular functions; its aberration leads to irregular cell proliferation and differentiation (i.e. pancreatic cancer). Previously, it was revealed that the formation of the complex of the 14-3-3 protein and the Son of sevenless homolog 1 (SOS1) - one of the main actors of the Ras-MAPK cascade -, would represent a key-process to downstream the deviant Ra-MAPK signaling. In this data article we attempt to shed some light on the 3D structure, providing useful details about the crystallization process of the 14-3-3ζ dimer in complex with the 13-mer SOS1pS^1161^. The crystal structure is deposited at the Protein Data Bank with identifier 6F08. This Data in Brief article refers to “Structural characterization of 14-3-3ζ in complex with the human Son of sevenless homolog 1 (SOS1) (2018).”

**Specifications Table**

**Table:** 

Subject area	Biological Chemistry
More specific subject area	Structural Biology
Type of data	Figures, movie, graphs
How data was acquired	X-Ray diffraction was performed at the Deutsches Elektronen-Synchrotron in Hamburg (Germany), Petra III, DESY beamline using a Detectris Pilatus 6 M detector. X-ray data was processed using iMOSFLM. The model was refined using both REFMAC and PHENIX software package and build using Coot.
Data format	Raw and analyzed
Experimental factors	None applied
Experimental features	Identification of crystal growth condition, crystal diffraction, crystal determination and refinement
Data source location	Eindhoven University of Technology, Eindhoven, The Netherlands
	Petra III, DESY beamline, Hamburg, Germany
Data accessibility	Crystallographic data within this article were deposited in the Protein Data Bank, PDB: 6F08.

**Value of the data**

• Provides the first crystal structure of 14-3-3 in complex with SOS1 binding partner
• Describes in detail the binding within the 14-3-3ζ dimer and SOS1-derived peptide
• This work could provide the structural basis for identifying new chemical compounds as the starting point for the development of therapeutic strategies in certain type of cancers

## 1 Data

We describe the crystallization and processing and the structure determination of the 14-3-3 ζ protein in complex with the SOS1-derivative peptide (1155-PRRRPE{pSer^1161^}APAESS-1167).

## 2 Experimental design, materials and methods

### 2.1 Crystallization and processing

**Fig. 1:**
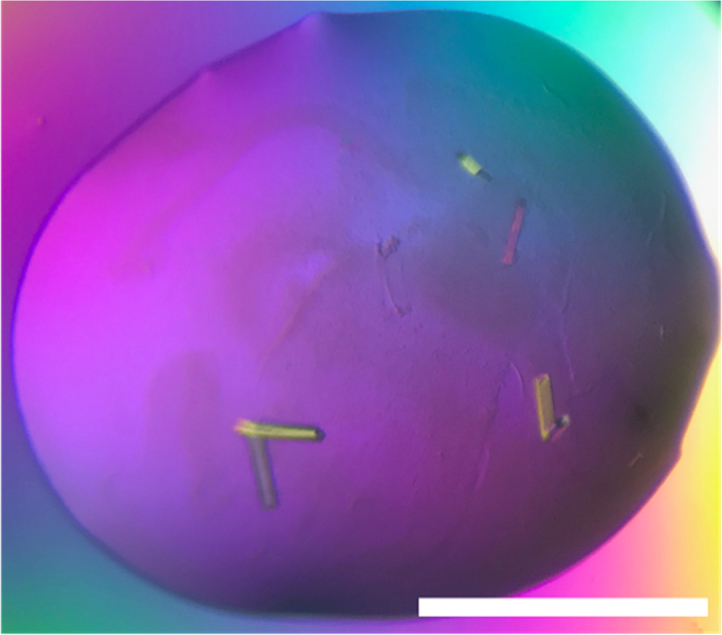
Photograph of the 14-3-3ζ/SOS1pSer^1161^ peptide complex crystals taken with a polarized light microscope. 14-3-3ζ/SOS1pSer^1161^ peptide grew as rounded plate shaped-crystals in presence of 0.1 M phosphate citrate pH 4.2, 36% (v/v) PEG 300 at room temperature. Scale bar derived from the diameter of the screw cap, it corresponds to 1 mm.

**Fig. 2:**
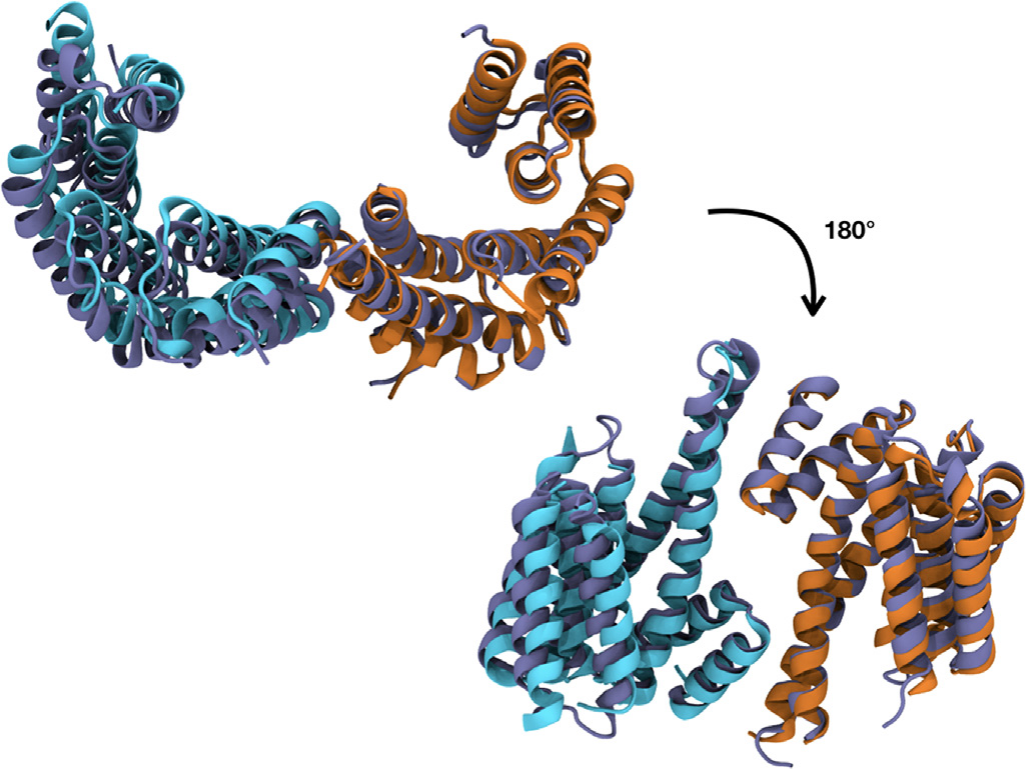
The superimposition between monomers A (orange) and B (cyan) of 6F08 and the model 1QJB (iceblue) emphasizes the C-terminal flexibility. The RMSD Tool Plugin from VMD [2] was used to calculate RMS (root mean square) distances between the backbone atoms of the two structures; the total RMSD is 1.749Å.

**Fig. 3:**
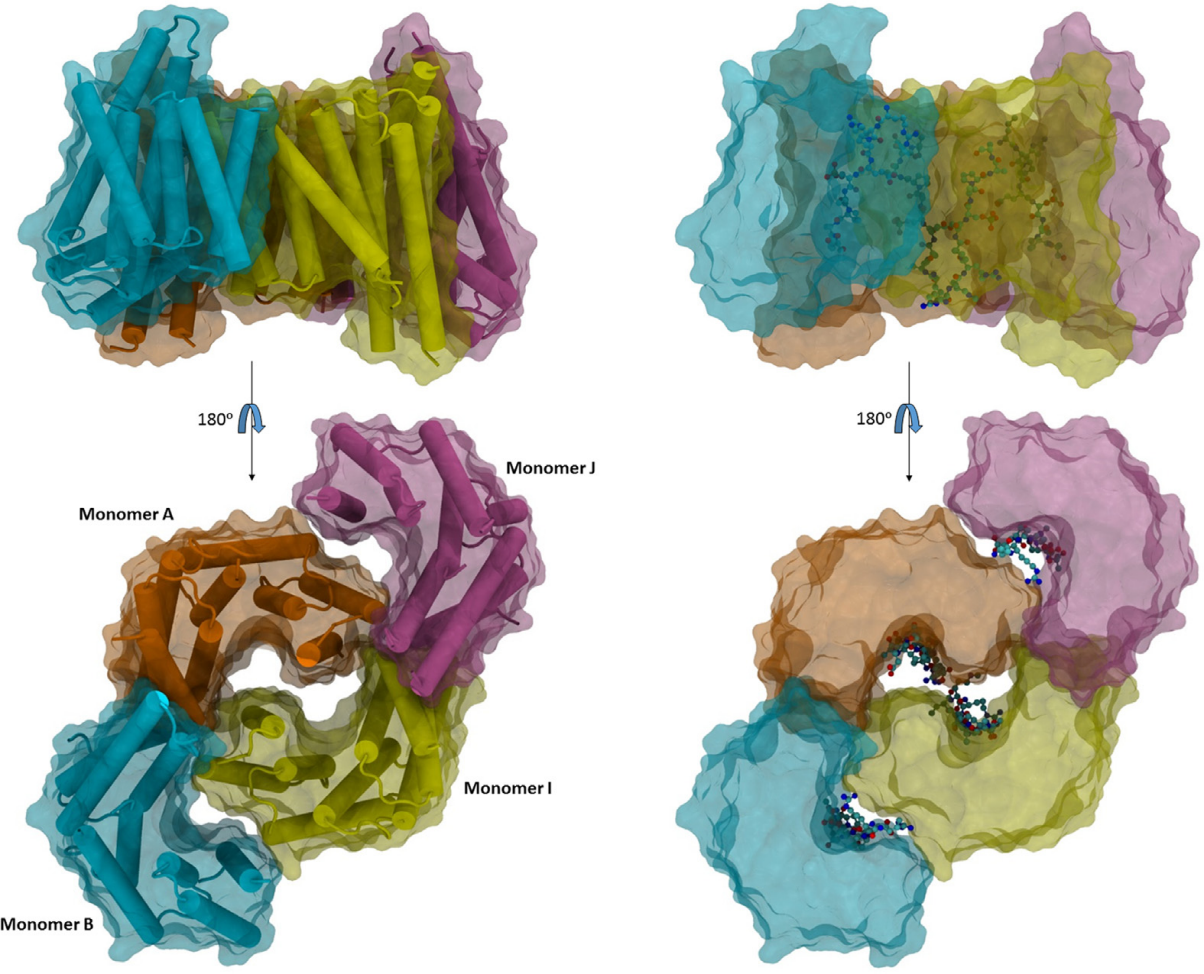
Crystal structure of SOS1pSer^1161^ peptide bound to 14-3-3ζ dimer. (A) Cartoon plot with the semitransparent surface of the 14-3-3ζ dimer. (B) Top view of the 14-3-3ζ dimer with the typical W-like shape; each monomer consists of a bundle of nine α-helices organized in an antiparallel fashion. (C) Surface plot of the 14-3-3ζ dimer bound to the SOS1pSer^1161^ (cyan rods); the peptides adopt an extended conformation. (D) Top view of the surface plot; all the phosphorylated peptides are lining the concave surface of the groove.

14-3-3ζ protein and SOS1pSer^1161^ peptide were mixed in a 1:2 M ratio to a final concentration of 10 mg mL^-1^ in 20 mM Hepes, 2 mM MgCl_2_, 2 mM DTT, pH 7.5; the complex was incubated at 4 °C overnight for crystallization. 14-3-3ζ/SOS1pSer^1161^ peptide was screened against the JCSG+ and JCSG from I to IV screens (Molecular Dimension) using a mosquito Crystal set up as 200 nL hanging drops at room temperature. The crystallization condition selected from the screens was 0.1 M phosphate citrate pH 4.2, 40% (v/v) PEG 300. The condition was optimized at 36% (v/v) PEG 300. Crystals grew within a week at room temperature and could be directly flash-cooled in mother liquor using liquid nitrogen. Flash frozen crystals were collected at Petra III DESY beamline (Hamburg, Germany). Diffraction data was processed using iMOSFLM [1] (Fig. 1, Fig. 2, Fig. 3).

### 2.2 Structure determination

The crystal of the heterodimeric 14-3-3ζ/ SOS1pSer^1161^ belonged to the space group P 1 21 1 with unit cell dimensions of a = 67.06 Å, b = 93.14 Å, c = 74.52 Å, α = 90.00 Å, β = 92.93 Å, γ = 90.00 Å. Crystals diffracted to 1.90 Å. Initial phase information were generated using PDB ID: 1QJB as the search model for molecular replacement (MR).

MR was performed using MOLREP [3]. The model was fully refined with no Ramachandran outliers using both REFMAC [4] and Phenix [5]. The structure model was deposited in the Protein Data Bank (accession code PDB ID: 6F08).

## Figures and Tables

**Video 1:**
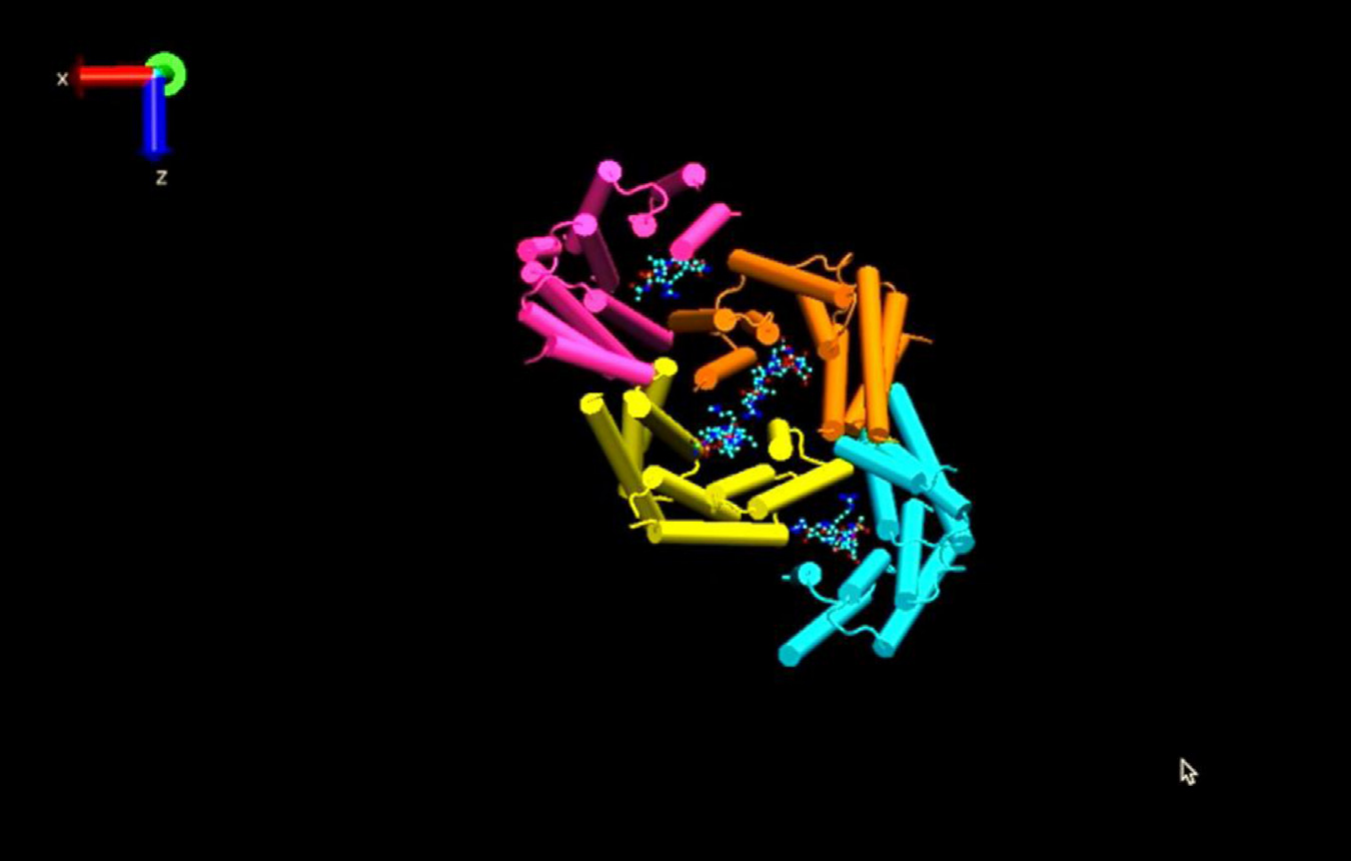
Crystal structure of 14-3-3ζ/ SOS1pSer^1161^ complex rotation (PDB ID: 6F08) [6]. A video clip is available online.
